# Defining Obesity Cut-Off Points for Migrant South Asians

**DOI:** 10.1371/journal.pone.0026464

**Published:** 2011-10-19

**Authors:** Laura J. Gray, Thomas Yates, Melanie J. Davies, Emer Brady, David R. Webb, Naveed Sattar, Kamlesh Khunti

**Affiliations:** 1 Department of Health Sciences, University of Leicester, Leicester, United Kingdom; 2 Department of Cardiovascular Sciences, University of Leicester, Leicester, United Kingdom; 3 Department of Diabetes Research, University Hospitals of Leicester, Leicester, United Kingdom; 4 Institute of Cardiovascular and Medical Sciences, BHF Glasgow Cardiovascular Research Centre, University of Glasgow, Glasgow, United Kingdom; German Diabetes Center, Leibniz Center for Diabetes Research at Heinrich Heine University, Germany

## Abstract

**Background:**

Body mass index (BMI) and waist circumference (WC) are used to define cardiovascular and type 2 diabetes risk. We aimed to derive appropriate BMI and WC obesity cut-off points in a migrant South Asian population.

**Methods:**

4688 White Europeans and 1333 South Asians resident in the UK aged 40–75 years inclusive were screened for type 2 diabetes. Principal components analysis was used to derive a glycaemia, lipid, and a blood pressure factor. Regression models for each factor, adjusted for age and stratified by sex, were used to identify BMI and WC cut-off points in South Asians that correspond to those defined for White Europeans.

**Findings:**

For South Asian males, derived BMI obesity cut-off points equivalent to 30.0 kg/m^2^ in White Europeans were 22.6 kg/m^2^ (95% Confidence Interval (95% CI) 20.7 kg/m^2^ to 24.5 kg/m^2^) for the glycaemia factor, 26.0 kg/m^2^ (95% CI 24.7 kg/m^2^ to 27.3 kg/m^2^) for the lipid factor, and 28.4 kg/m^2^ (95% CI 26.5 kg/m^2^ to 30.4 kg/m^2^) for the blood pressure factor. For WC, derived cut-off points for South Asian males equivalent to 102 cm in White Europeans were 83.8 cm (95% CI 79.3 cm to 88.2 cm) for the glycaemia factor, 91.4 cm (95% CI 86.9 cm to 95.8 cm) for the lipid factor, and 99.3 cm (95% CI 93.3 cm to 105.2 cm) for the blood pressure factor. Lower ethnicity cut-off points were seen for females for both BMI and WC.

**Conclusions:**

Substantially lower obesity cut-off points are needed in South Asians to detect an equivalent level of dysglycemia and dyslipidemia as observed in White Europeans. South Asian ethnicity could be considered as a similar level of risk as obesity (in White Europeans) for the development of type 2 diabetes.

## Introduction

Excess adiposity is a key risk factor for many chronic diseases, the most prevalent of which are type 2 diabetes and cardiovascular disease (CVD), collectively called cardiometabolic disease [1], and is often used to identify high risk groups. Body mass index (BMI), calculated as weight (kg) divided by height squared (m^2^), is the most common method of assessing adiposity in usual health care practice. Based on epidemiological evidence investigating associations with mortality and morbidity, BMI cut-off points of 25 kg/m^2^ and 30 kg/m^2^ are conventionally used to classify overweight and obesity respectively [1]. Whilst most of the evidence underpinning these BMI cut-off points originates from White populations, there is increasing evidence that levels of risk associated with the classification of overweight and obesity vary across racial groups [2]. In 2004, an expert consultation by the World Health Organization (WHO) recommended revised BMI cut-off points for the classification of overweight (23 kg/m^2^) and obesity (27.5 kg/m^2^) in Asian populations [3]. However, further evidence suggests that these revised categorisations may still under represent the risk of cardiometabolic disease in people originating from India, Pakistan and Bangladesh [4]–[7]. A recent consensus statement advised that BMI cut-off points for overweight and obesity should be lowered to 23 kg/m^2^ and 25 kg/m^2^ respectively for Indian Asians [8], this was reiterated by The South Asian Health Foundation, UK [9]. This has major implications because health care organisations increasingly advocate the use of obesity prevention and treatment as a method of reducing the risk of cardiometabolic disease. There has therefore been a call for determining obesity thresholds from further scientific evidence in minority populations [10].

Waist circumference (WC) is another clinically relevant and increasingly used method of assessing adiposity. Whereas BMI provides a marker of overall adiposity, WC provides a surrogate marker of abdominal adiposity, and is the best correlate to visceral fat mass. Higher levels of WC are strongly associated with risk factors for, and incidence of, cardiometabolic disease and the relationships to some outcomes (in particular diabetes in women) have been shown to be independent of BMI [11]–[15]. WHO currently recommends WC cut-off points for identifying those with an increased and a substantially increased risk of having or developing a chronic disease of 80 cm and 88 cm respectively for women and 94 cm and 102 cm respectively for men of European extraction [1]. However, as with BMI, population specific cut-off points for WC are needed. Recent recommendations from expert groups in the UK and India advise that a WC cut-off point of 80 cm for women and 90 cm for men should be used to identify obesity in South Asians [8], [9]. It has also been suggested that lifestyle advice should be sought by South Asian women with a WC greater than 72 cm and South Asian men with a WC greater than 78 cm [8]. However these latter recommendations are based on limited data and require further research.

The aim of this paper was to derive appropriate BMI and WC cut-off points for a migrant South Asian population whereby classification of obesity are associated with similar levels of dysglycemia, dyslipidemia and hypertension as those seen in a White European population.

## Methods

### Ethical approval

Approval was obtained from the University Hospitals of Leicester (UHL09320) and Leicestershire Primary Care Research Alliance (64/2004) local research ethics committees for the Leicester addition study. No ethical approval was required for the analysis carried out in this manuscript.

### Participants

This study uses baseline data from the Leicester (UK) Addition study, a population based screening programme. This study has been described in detail elsewhere [16]. Briefly, 20 local community practices from Leicester participated in a screening programme inviting a random sample of people between 40–75 years inclusive (25–75 years for South Asians) to attend a single session glucose and cardiovascular risk assessment. Those with known diabetes were excluded. The screening phase ran from August 2004 to December 2007 and screened 6,749 individuals from the 30,950 invited (response rate 22%). Written informed consent was obtained from all participants. Consistent with national census data, participants were asked to classify themselves into one of 16 ethnic groups; these included three categories of White (British, Irish, or other), four categories of Asian or Asian British (Indian, Pakistani, Bangladeshi, or other) and nine other categories (4 mixed race categories, Chinese, Chinese other, Caribbean, African, or any other black background). This study reports data only from those classifying themselves as White or Asian, as other groups were too small to provide meaningful data. Since the South Asian sub groups were predominantly of Indian origin and also have broadly common ancestry, we combined them into one ethnic group. In order to limit differences in age between ethnicities we excluded South Asians younger than 40 years of age (n = 331).

### Biochemical, clinical and demographic measurements

Venous blood samples were collected in the morning following an overnight fast. All individuals underwent a standardised oral glucose tolerance test [17]. Blood lipids levels, fasting glucose, two hour glucose, and HbA1c were measured in the same laboratory located within Leicester Royal Infirmary, UK, using stable methodology standardised to external quality assurance reference values. Glucose samples were taken in fluoride oxalate test tubes and placed immediately in a portable 4 litre 4°C refrigerator (also available on board the mobile screening unit). HbA1c was analysed by a DCCT aligned Biorad Variant HPLC II system (Bio-Rad laboratories, Hemel Hempstead, UK). The imprecision coefficient of variation of this machinery was <0.1%, the reference intervals fit with national recommendations valid for carriers of variant Hb S, C and Q. Samples were processed within a maximum of two hours, using an Abbott Aeroset clinical chemistry analyser (Abbott laboratories, Maidenhead, UK), which employs the hexokinase enzymatic method. This machinery has an imprecision coefficient of variation of 1.61%. Serum total cholesterol, high-density lipoprotein (HDL) cholesterol, low-density lipoprotein (LDL) cholesterol, and triglycerides were measured by means of enzymatic techniques (Dade Behring Dimension analyser, Newark, USA). Type 2 diabetes was diagnosed using WHO 1999 criteria [17].

Arterial blood pressure was measured in the sitting position (Omron Healthcare, Milton Keynes, UK); three measurements were obtained and the average of the last two measurements was used. Body weight (Seca UK, Birmingham, UK), WC (midpoint between the lower costal margin and iliac crest) and height were also measured to the nearest 0.1 kg and 0.5 cm respectively, by personnel trained in the ascertainment of these measures.

### Statistical analysis

All analyses were carried out using Stata (Version 10.0). Baseline characteristics for White Europeans and South Asians male and females were presented as mean (standard deviation) for continuous variables and count (percentage) for categorical ones. Differences between White Europeans and South Asians were calculated using the t-test for continuous data and chi square test for categorical. The relationship between BMI and WC and each risk factor of interest was assessed using linear regression, an interaction term was used to asses ethnicity related differences between BMI and WC and the risk factors.

Principal components analysis was used to derive a single summary variable for each measured group of cardiovascular risk factors. The glycaemia factor combined fasting and 2 hour glucose, and HbA1c, the lipid factor combined HDL and triglycerides and the blood pressure factor combined systolic and diastolic blood pressure. Regression models were fitted with each factor as the dependent variables separately for males and females, all models contained BMI, ethnicity and an interaction between BMI and ethnicity. All models were adjusted for age centred at the mean. The predicted value for each factor was then calculated for a BMI 30 kg/m^2^ for White Europeans with mean age. We then determined the value of BMI which gave the same predicted values for South Asians to give equivalent cut-off points. 95% confidence intervals (95% CI) were estimated from the 95% confidence bands from the predicted values. This process was then repeated using WC. 102 cm was used to define those at a substantially increased risk of obesity related complications in White European males, and 88 cm was used for White European females. For each model fitted the assumptions were assessed: checking the residuals for normality and homoscedasticity and the linearity of the relationship between the outcome and the predictors.

Sensitivity analyses were carried out. Firstly we repeated the modelling excluding those who are taking lipid lowering medication from the lipid analysis and those who are taking antihypertensive medication from the blood pressure analysis. Secondly we excluded HbA1c from the glycaemia factor analysis as studies have shown ethnic differences for HbA1c that are independent of fasting and 2 hour glucose measurements [18], [19].

## Results

Six thousand and forty one participants were included (Table 1). The South Asians were on average 5.6 years younger than the White Europeans (58.6 years versus 53.0 years, p<0.0001), South Asian males were significantly older than South Asian females (p = 0.0004), no difference between sexes was found for White Europeans (p = 0.39). South Asian males had a lower mean BMI than South Asian females and the White Europeans. South Asians had higher 2 hour glucose and HbA1c compared to White Europeans. The prevelence of glucose disorders were higher in the South Asians, but they have lower rates of dyslipidemia and hypertension. Statistically significant linear relationships were found between BMI and WC and all of the risk factors of interest (fasting glucose, 2 hour glucose, HbA1c, HDL cholesterol, triglycerides and systolic and diastolic blood pressure). Statistically significant ethnic differences were seen for the relationships between BMI with HDL cholesterol and triglycerides and between WC with 2 hour glucose, systolic blood pressure and HDL cholesterol (test for BMI*ethnicity and WC*ethnicity interactions p<0.05) (Tables S1 and S2).

**Table 1:** Baseline characteristics.

	South Asian (SA)			White European (WE)			Total	SA vs. WE
	Total SA	Males	Females	Total WE	Males	Females		P value
		665	688		2209	2479	6041	
Age(years)	53.0 (8.7)	53.9 (9.1)	52.2 (8.2)	58.6 (9.5)	58.7 (9.5)	58.5 (9.5)	57.3 (9.6)	<0.0001
BMI (kg/m^2^)	27.5 (4.9)	26.6 (4.1)	28.4 (5.4)	28.3 (5.0)	28.2 (4.2)	28.4 (5.6)	28.1 (5.0)	<0.0001
<18.5 kg/m^2^, n (%)	19 (1.4)	12 (1.8)	7 (1.0)	18 (0.4)	1 (0.1)	17 (0.7)	37 (0.6)	<0.0001†
18.5 kg/m^2^–<25 kg/m^2^, n (%)	411 (30.4)	225 (33.9)	186 (27.1)	1180 (25.3)	458 (20.9)	722 (29.3)	1591 (26.5)	
25 kg/m^2^–<30 kg/m^2^, n (%)	571 (42.3)	310 (46.7)	261 (28.0)	2021 (43.4)	1104 (50.4)	917 (37.2)	2592 (42.1)	
> = 30 kg/m^2^, n (%)	350 (25.9)	117 (17.6)	233 (33.9)	1440 (30.9)	628 (28.7)	812 (32.9)	1790 (29.8)	
Waist (cm)	92.6 (11.6)	95.8 (10.2)	89.6 (12.2)	94.7 (13.5)	100.0 (11.4)	89.9 (13.4)	94.2 (13.1)	<0.0001
Systolic BP (mmHg)	134.8 (19.1)	138.6 (18.0)	131.2 (19.5)	139.0 (19.4)	141.8 (18.0)	136.4 (20.2)	138.0 (19.4)	<0.0001
Diastolic BP(mmHg)	85.6 (10.8)	86.8 (10.6)	84.4 (10.9)	85.7 (10.5)	87.1 (10.1)	84.4 (10.6)	85.7 (10.5)	0.75
Total cholesterol (mmol/l)	5.2 (1.0)	5.2 (1.0)	5.2 (0.9)	5.7 (1.1)	5.5 (1.1)	5.8 (1.1)	5.6 (1.1)	<0.0001
LDL-cholesterol (mmol/l)	3.3 (0.8)	3.4 (0.9)	3.3 (0.8)	3.6 (0.9)	3.6 (0.9)	3.7 (0.9)	3.6 (0.9)	<0.0001
HDL-cholesterol (mmol/l)	1.2 (0.3)	1.2 (0.3)	1.3 (0.3)	1.4 (0.4)	1.3 (0.3)	1.5 (0.4)	1.4 (0.4)	<0.0001
Triglycerides (mmol/l)	1.4 (0.8)	1.6 (0.9)	1.3 (0.8)	1.4 (0.9)	1.6 (1.1)	1.3 (0.7)	1.4 (0.9)	0.73
Fasting glucose (mmol/l)	5.3 (0.9)	5.4 (0.9)	5.1 (0.8)	5.2 (0.9)	5.3 (1.1)	5.1 (0.8)	5.2 (0.9)	0.002
2 hour glucose (mmol/l)	6.5 (2.7)	6.5 (2.8)	6.4 (2.5)	5.9 (2.4)	5.8 (2.6)	5.9 (2.2)	6.0 (2.5)	<0.0001
HbA1c (%)	5.9 (0.6)	5.9 (0.7)	5.8 (0.6)	5.7 (0.6)	5.7 (0.7)	5.6 (0.5)	5.7 (0.6)	<0.0001
Type 2 Diabetes, n (%)	68 (5.1)	40 (6.1)	28 (4.1)	128 (2.7)	75 (3.4)	53 (2.2)	196 (3.3)	<0.0001
IGT, n (%)	198 (14.7)	100 (15.2)	98 (14.3)	505 (10.8)	224 (10.2)	281 (11.4)	703 (11.7)	<0.0001
IFG, n (%)	69 (5.1)	43 (6.5)	26 (3.8)	206 (4.4)	114 (5.2)	92 (3.7)	275 (4.6)	0.27
Hypertension, n (%)	604 (45.1)	329 (50.0)	275 (40.3)	2390 (51.8)	1252 (57.4)	1138 (46.8)	2994 (50.3)	<0.0001
Dyslipidemia, n (%)	792 (59.2)	390 (59.4)	402 (59.1)	3418 (73.6)	1508 (68.9)	1910 (77.9)	4210 (70.4)	<0.0001
Antihypertensive therapy, n (%)	297 (22.0)	162 (24.4)	135 (19.6)	1182 (25.2)	572 (25.9)	610 (24.6)	1479 (24.5)	0.01
Lipid lowering therapy, n (%)	143 (10.6)	95 (14.3)	48 (7.0)	576 (12.3)	328 (14.9)	248 (10.0)	719 (11.9)	0.09

Data given as mean (standard deviation) unless stated. Comparison of South Asians and White Europeans is made using t-test for continuous variables and the chi square test for categorical.

IGT: Impaired glucose tolerance, IFG: Impaired fasting glucose, hypertension defined as systolic > = 140 mmHg or diastolic > = 90 mmHg, dyslipidemia defined as total cholesterol > = 5.0 mmol/l,

† Chi square test compares 4 BMI groups by ethnicity.

### BMI cut-off points

**Figure 1:**
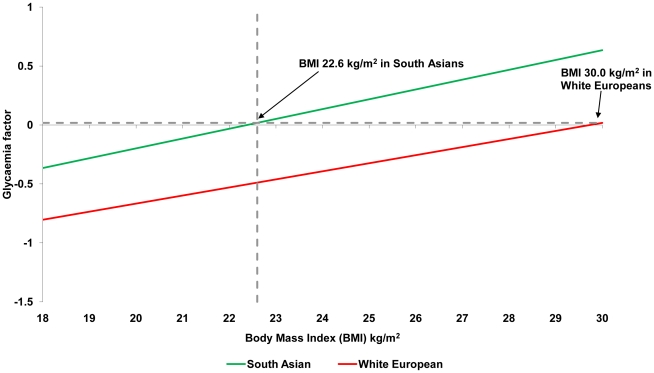
Relationship between glycaemia factor and BMI among White European and South Asian males. The glycaemia factor is the single summary variable derived from the principal components analysis using fasting glucose, 2 hour glucose and HbA1c.

**Figure 2:**
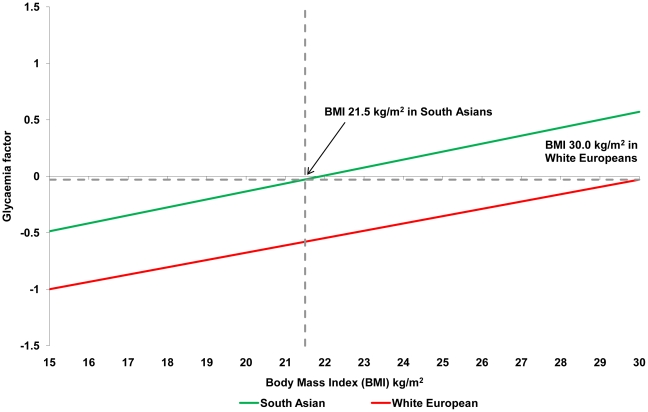
Relationship between glycaemia factor and BMI among White European and South Asian females. The glycaemia factor is the single summary variable derived from the principal components analysis using fasting glucose, 2 hour glucose and HbA1c.

Table 2 shows the equivalent BMI obesity cut-off points for South Asians compared to White Europeans. For a BMI of 30 kg/m^2^ in White Europeans cut-off points for South Asian males were: glycaemia 22.6 kg/m^2^ (20.7 kg/m^2^ to 24.5 kg/m^2^); lipid 26.0 kg/m^2^ (24.7 kg/m^2^ to 27.3 kg/m^2^); blood pressure 28.4 kg/m^2^ (26.5 kg/m^2^ to 30.4 kg/m^2^). Similar obesity cut-off points were identified for females: glycaemia 21.5 kg/m^2^ (19.5 kg/m^2^ to 23.5 kg/m^2^); lipid 23.9 kg/m^2^ (22.0 kg/m^2^ to 25.7 kg/m^2^); blood pressure 29.1 kg/m^2^ (24.9 kg/m^2^ to 33.3 kg/m^2^). Figures 1 and 2 demonstrate how these cut-off points were calculated for the glycaemia factor in males and females. The figures for the lipid and blood pressure factors are given in the supporting information (Figures S1, S2, S3, S4).

**Table 2:** White European equivalent cut-off point for South Asians for a BMI of 30 kg/m^2^.

	Males	Females
Glycaemia factor	22.6 kg/m^2^ (20.7 kg/m^2^ to 24.5 kg/m^2^)	21.5 kg/m^2^ (19.5 kg/m^2^ to 23.5 kg/m^2^)
Lipid factor	26>0 kg/m^2^ (24.7 kg/m^2^ to 27.3 kg/m^2^)	23.9 kg/m^2^ (22.0 kg/m^2^ to 25.7 kg/m^2^)
Blood pressure factor	28.4 kg/m^2^ (26.5 kg/m^2^ to 30.4 kg/m^2^)	29.1 kg/m^2^ (24.9 kg/m^2^ to 33.3 kg/m^2^)

Factor is the single summary variable derived from the principal components analysis. Glycaemia factor includes fasting glucose, 2 hour glucose and HbA1c; Lipid factor includes HDL cholesterol and triglycerides; Blood pressure factor includes systolic and diastolic blood pressure.

### Waist circumference cut-off points

Lower cut-off points were seen for South Asians. South Asian cut-off points ranged from 83.8 cm (79.3 cm to 88.2 cm) to 99.3 cm (93.5 cm to 105.2 cm) in men and from 69.3 cm (65.2 cm to 73.4 cm) to 86.6 cm (77.4 cm to 95.8 cm) in women (Table 3).

**Table 3:** White European equivalent waist circumference (cm) cut-off points for South Asians.

	Males	Females
	(White European = 102 cm)	(White European = 88 cm)
Glycaemia factor	83.8 cm (79.3 cm to 88.2 cm)	69.3 cm (65.2 cm to 73.4 cm)
Lipid factor	91>4 cm (86.9 cm to 95.8 cm)	74.2 cm (69.3 cm to 79.0 cm)
Blood pressure factor	99.3 cm (93.3 cm to 105.2 cm)	86.6 cm (77.4 cm to 95.8 cm)

Factor is the single summary variable derived from the principal components analysis. Glycaemia factor includes fasting glucose, 2 hour glucose and HbA1c; Lipid factor includes HDL cholesterol and triglycerides; Blood pressure factor includes systolic and diastolic blood pressure.

### Sensitivity analysis and assumptions

Excluding those on antihypertensive (1,479 (24.5%)) or lipid lowering (719 (11.9%)) medication gave comparable results for both BMI (Table S3) and WC (Table S4). When excluding HbA1c from the glycaemia factor, slightly higher cut-off points were seen for both BMI and WC for males and females (Tables S5 and S6). The statistical assumptions were satisfied for all of the models fitted.

## Discussion

Using data from a large diabetes screening study, we used principal components analysis to combine multiple cardiometabolic risk markers into single glycaemia, lipid, and blood pressure factors. For example, the derived glycaemia factor, calculated obesity cut-off points for South Asian were 22.6 kg/m^2^ for males and 21.5 kg/m^2^ for females. WC cut-off points were also substantially lower in South Asians than those currently recommended [1].

It has consistently been shown that the prevalence of many chronic diseases in developed countries is substantially higher in South Asian populations, for example the risk of type 2 diabetes is between 2 and 6 times higher in South Asians compared to White Europeans [20]–[22]. However there has been little attempt to investigate whether ethnic differences exist in the association between levels of adiposity and cardiometabolic risk. To our knowledge, only one other modest study (n = 590) has directly compared BMI cut-off points for obesity between migrant South Asians and White Europeans [4]. Using similar methodology as applied here, they reported BMI cut-off points for obesity of between 21.1 kg/m^2^ and 22.5 kg/m^2^ in South Asians [4]. Studies using receiver operating characteristic curves methodology for identifying optimal BMI cut-off points for discriminating between those with and without type 2 diabetes or the metabolic syndrome have shown cut-off points between 22 kg/m^2^ and 23 kg/m^2^ [5], [6], [23]. While a cut-off point of 21 kg/m^2^ has been reported to be optimal for identifying the presence of at least one CVD risk factor [7]. It has also been suggested that ethnic specific cut-off points may vary by country [24]. In contrast a Canadian study found that cut-off points should be lowered for East Asians but not for South Asians, however this study was carried out on a limited sample (n = 357) [25]. A recent large study (n = 7729) looking at cut-off points for WC only for White Europeans and South Asians also concurred that lower cut-off points are needed to define equivalent levels of diabetes risk [26]. The use of principal components analysis to combine multiple risk markers into a single factor has several advantages over other methods, such as receiver operating characteristic curves or logistic regression, these have been described in detail elsewhere [4]. No other studies have derived both BMI and WC cut-off points using this methodology on a large bi ethnic population based UK data set.

These results highlight the fact that South Asians face the double burden of dysglycemia and dyslipidemia at substantially lower levels of adiposity than White Europeans; this has some important implications. In order to ensure equivalence in targeting at risk individuals, BMI and WC cut-off points need to be substantially lower in South Asian populations than those currently recommended by WHO. Indeed, for most of the factors considered, the majority of the South Asian population in developed countries have a BMI over the obesity BMI cut-points suggested here. For example, the majority of South Asian participants in this population based study of 40–75 year olds had a BMI of over 25 kg/m^2^. Therefore, the vast majority of South Asians will have a higher risk of cardiometabolic disease, particularly type 2 diabetes, than a White European with a BMI of 30 kg/m^2^. Consequently South Asian ethnicity per se should be viewed as denoting a similar level of risk as obesity does in White Europeans.

This data should not be used to infer that South Asians with a BMI of less than 22.6 kg/m^2^, have excess adiposity and hence require weight loss intervention; however it does suggest that in addition to traditional obesity management initiatives, other prevention strategies and educational messages urgently need to be developed, evaluated and promoted in this population. For example, South Asians have consistently been found to be substantially less physically active than White European populations in industrialised countries [27] and intervention studies aimed at the promotion of physical activity have been shown to be effective at targeting/attenuating cardiometabolic risk factors across many different population groups, including South Asians [28]. However, examples of multifactorial health behaviour interventions that have been developed and evaluated in migrant South Asian populations are lacking.

This study did not observe differences between South Asians and White Europeans in the distribution of blood pressure across levels of BMI or WC. This finding confirms other smaller studies in multi ethnic populations and suggests that hypertension per se does not appear to contribute to the increased risk of cardiometabolic disease observed in South Asians over White Europeans [4], [29].

### Study limitations

Although this is the largest study to date to derive cut-off points for migrant South Asians using robust methodology, there are some limitations which need to be taken into account when interpreting the data. Firstly, the cross sectional design limited our analysis to risk factors for, rather than the incidence of, chronic disease. Whilst it is clear that elevated levels of glycaemia and lipids are key risk factors for type 2 diabetes and CVD, our analysis assumed that these risk markers are equally predictive of future chronic disease across ethnicities. Whilst there are little published data to confirm or reject this assumption, evidence from a subset of participants included in the present study found that South Asians with impaired glucose tolerance and/or impaired fasting glucose were almost 3 times more likely to progress to type 2 diabetes than White Europeans with the same diagnosis over a 12 month period [30], consistent with a greater metabolic susceptibility to weight gain. This suggests that the calculated South Asian BMI cut-off points for obesity derived from the glycaemia factor were in fact a conservative interpretation of the data. Secondly, 22% of those invited came forward for screening; this is similar to, or higher than, other studies in similar populations [31] and reflects the difficulty in recruiting a multi-ethnic urban population with wide variations in social economic status into clinical trials. Additionally, those screened were slightly older than those invited [32]. Furthermore, the difference in sample size between the South Asian and White European cohorts (1,353 and 4,688 respectively) and the differences seen between these groups in terms of their risk profile may impact the power of this analysis. We recognise that although these limitations may compromise the representativeness of the sample, the large size of the cohort and the high quality of the collected data increase the credibility of our findings. Thirdly, ethnicity was recorded by asking the participants to classify themselves into one of 16 ethnic groups. Ancestry was not considered in this classification. In deriving the cut-off points we were not able to take into account environmental factors such as nutrition which may be important, especially in a migrant population. Therefore the results should not be generalized to all South Asian populations. Finally the derived factors for glycaemia, lipids and blood pressure should only be interpreted as crude summaries of these components. They do not include all possible variables which could be related to these factors, but rather those that were related to BMI and WC. As seen in the previous study using this methodology, not all of the variation was explained by the variables included in the three factors [4].

### Summary

Substantially lower obesity cut-off points are needed in South Asians to detect an equivalent level of dysglycemia in particular and dyslipidemia as observed in White Europeans. As the majority of South Asians have a BMI above the obesity cut-off point derived from the glycaemia factor, South Asian ethnicity per se could be viewed as conferring a similar level of risk for developing type 2 diabetes as obesity does in White Europeans. Twenty percent of the total world population is of South Asian ethnicity and therefore it is important that South Asians are offered the same treatment at an equivalent level of risk to other ethnicities. Our data also suggest a need to strongly reiterate that South Asians have an even greater need to maintain a healthy weight than the European Whites.

## Supporting Information

Figure S1 Relationship between lipid factor and BMI among White European and South Asian males. The lipid factor is the single summary variable derived from the principal components analysis using HDL cholesterol and triglycerides.
(TIF)

Figure S2 Relationship between lipid factor and BMI among White European and South Asian females. The lipid factor is the single summary variable derived from the principal components analysis using HDL cholesterol and triglycerides.
(TIF)

Figure S3 Relationship between blood pressure factor and BMI among White European and South Asian males. The blood pressure factor is the single summary variable derived from the principal components analysis using systolic and diastolic blood pressure.
(TIF)

Figure S4 Relationship between blood pressure factor and BMI among White European and South Asian females. The blood pressure factor is the single summary variable derived from the principal components analysis using systolic and diastolic blood pressure.
(TIF)

Table S1 Relationship between BMI (kg/m^2^) and risk factors.
(DOC)

Table S2 Relationship between waist circumference (cm) and risk factors.
(DOC)

Table S3 White European equivalent cut-off point for South Asians for a BMI of 30 kg/m^2^ excluding those on antihypertensive or lipid lowering medication.
(DOC)

Table S4 White European equivalent waist circumference (cm) cut-off points for South Asians excluding those on antihypertensive or lipid lowering medication.
(DOC)

Table S5 White European equivalent cut-off point for South Asians for a BMI of 30 kg/m^2^ excluding HbA1c from the glycaemia factor.
(DOC)

Table S6 White European equivalent waist circumference (cm) cut-off points for South Asians excluding HbA1c from the glycaemia factor.
(DOC)
