# Supplementary figures and images for: Defining Obesity Cut-Off Points for Migrant South Asians
Source: PLoS One. 2011 Oct 19;6(10):e26464. doi: 10.1371/journal.pone.0026464 (PMC3198431; doi:10.1371/journal.pone.0026464)

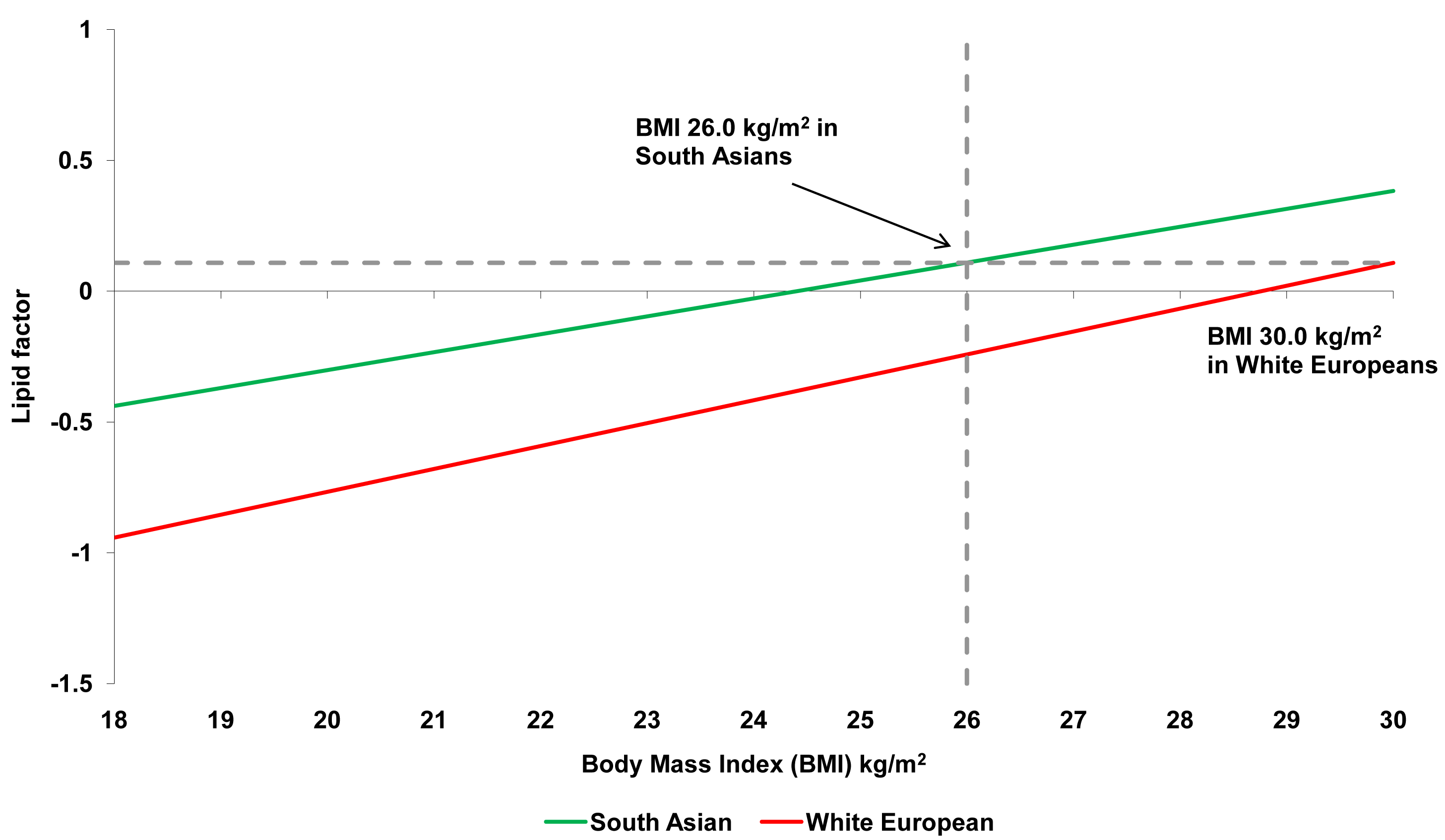

Supplement: Figure S1 — Relationship between lipid factor and BMI among White European and South Asian males. The lipid factor is the single summary variable derived from the principal components analysis using HDL cholesterol and triglycerides. (TIF) [file pone.0026464.s001.tif]

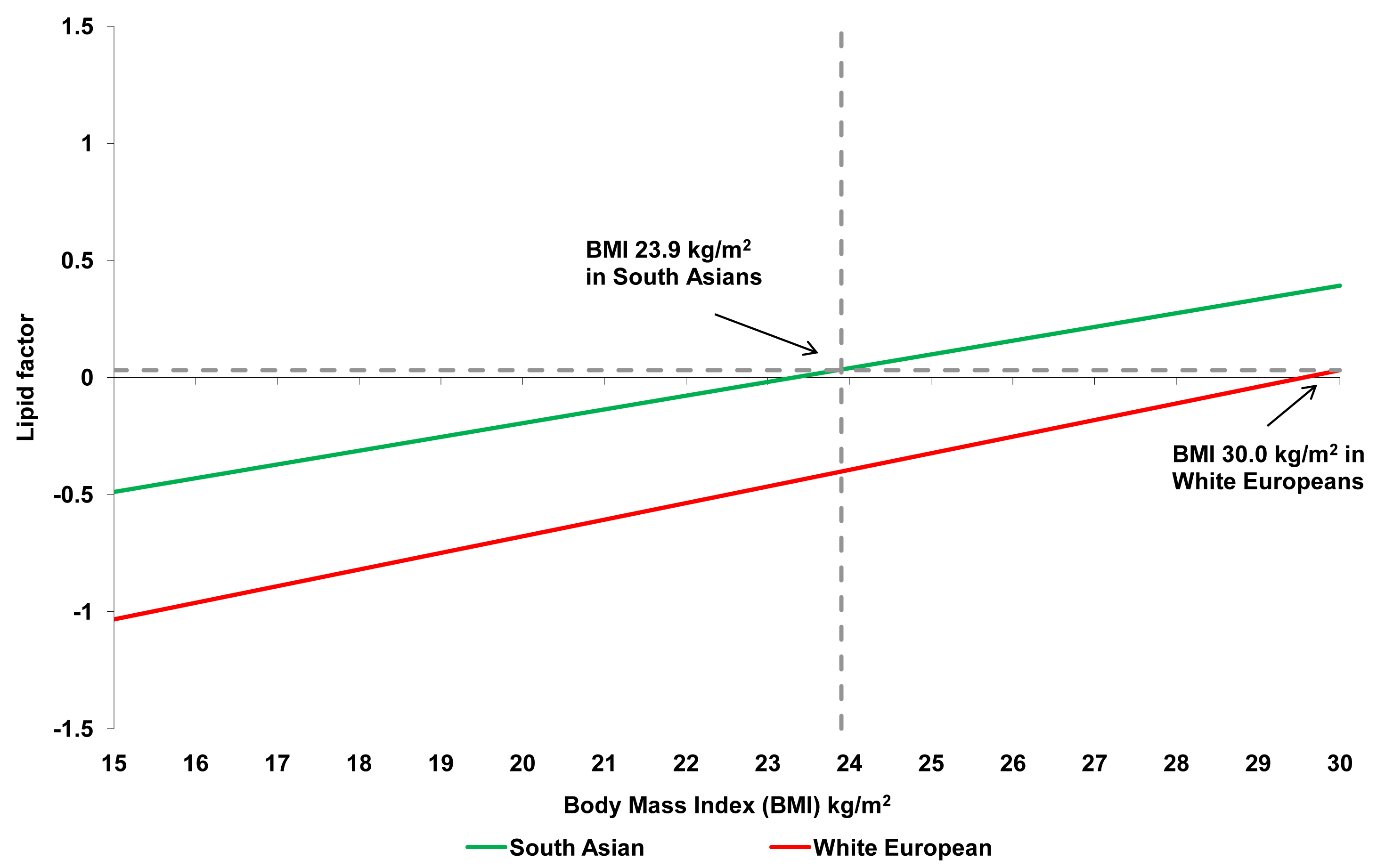

Supplement: Figure S2 — Relationship between lipid factor and BMI among White European and South Asian females. The lipid factor is the single summary variable derived from the principal components analysis using HDL cholesterol and triglycerides. (TIF) [file pone.0026464.s002.tif]

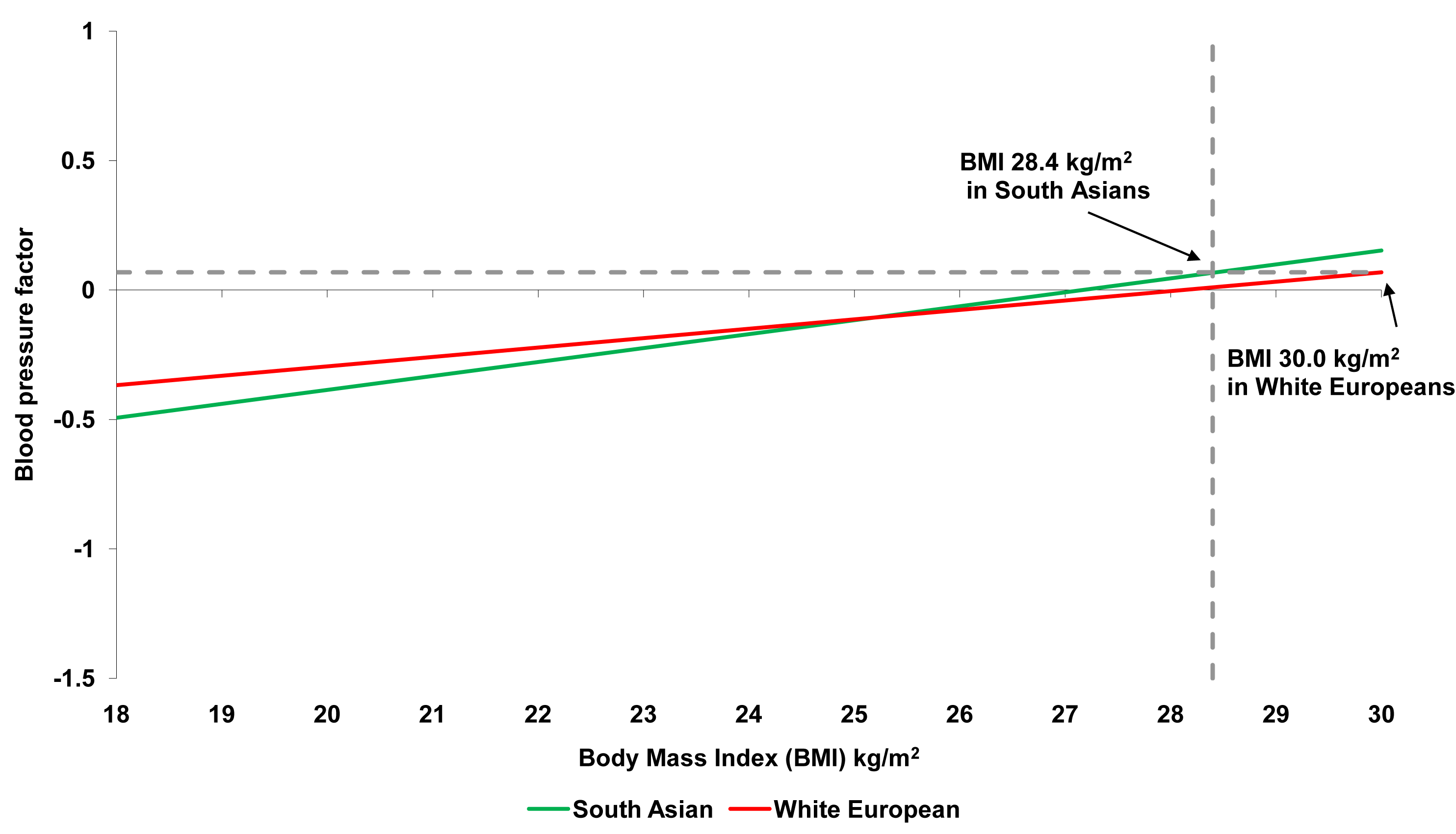

Supplement: Figure S3 — Relationship between blood pressure factor and BMI among White European and South Asian males. The blood pressure factor is the single summary variable derived from the principal components analysis using systolic and diastolic blood pressure. (TIF) [file pone.0026464.s003.tif]

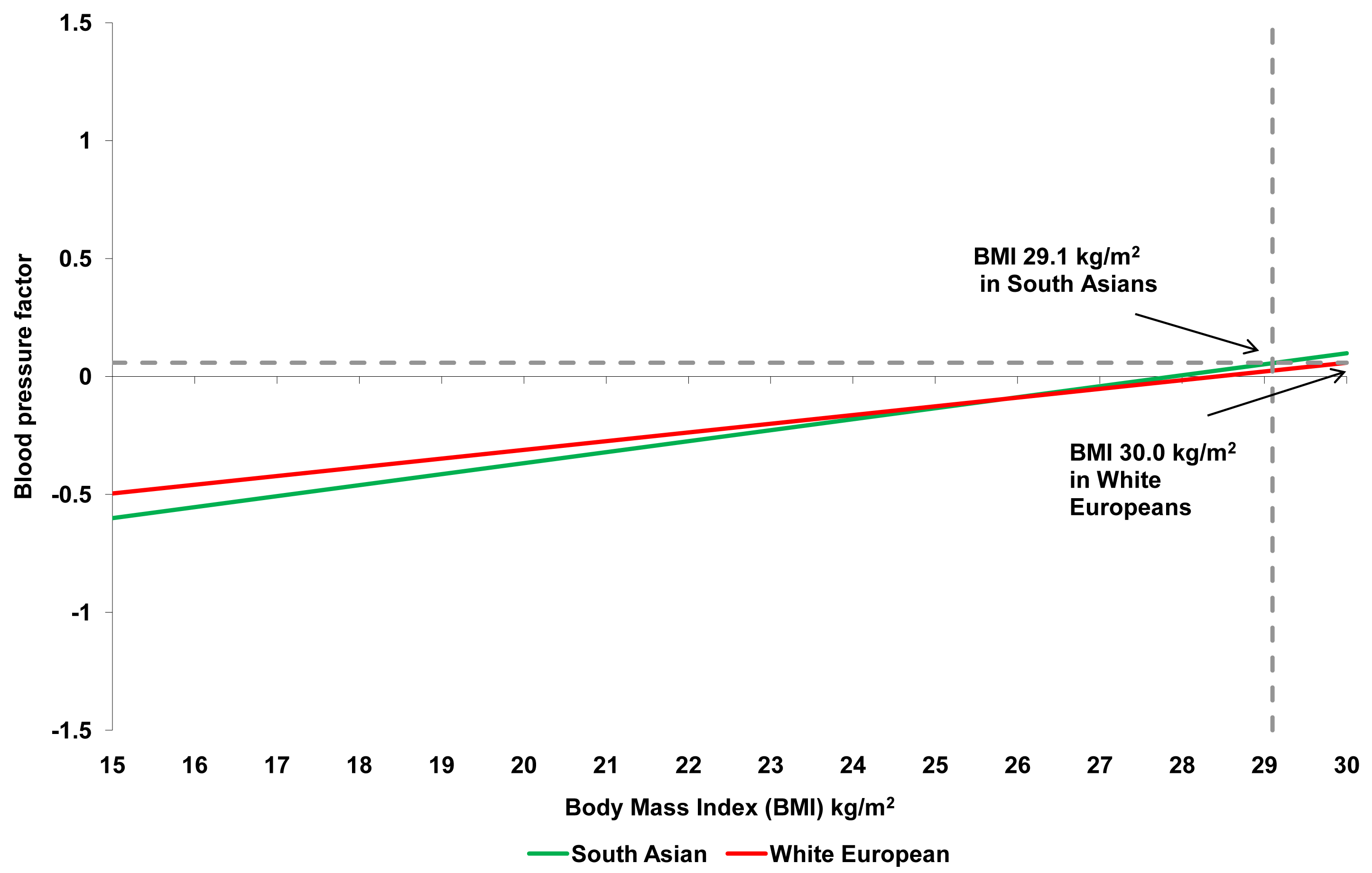

Supplement: Figure S4 — Relationship between blood pressure factor and BMI among White European and South Asian females. The blood pressure factor is the single summary variable derived from the principal components analysis using systolic and diastolic blood pressure. (TIF) [file pone.0026464.s004.tif]
