# Supplementary material for: Defining Obesity Cut-Off Points for Migrant South Asians
Source: PLoS One. 2011 Oct 19;6(10):e26464. doi: 10.1371/journal.pone.0026464 (PMC3198431; doi:10.1371/journal.pone.0026464)
Supplement: Table S1 — Relationship between BMI (kg/m2) and risk factors. (DOC) [file pone.0026464.s005.doc]

**Table S1**

**Relationship between BMI (kg/m^2^) and risk factors**

|  | Total cohort | | | Ethnicity interaction (effect of South Asian ethnicity compared to White European) | | |
| --- | --- | --- | --- | --- | --- | --- |
|  | Coefficient | 95% Confidence Interval | P value | Coefficient | 95% Confidence Interval | P value |
| Fasting glucose (mmol/l) | 0.03 | 0.03 to 0.04 | <0.0001 | -0.01 | -0.02 to 0.002 | 0.15 |
| 2 hour glucose (mmol/l) | 0.09 | 0.08 to 0.10 | <0.0001 | 0.01 | -0.02 to 0.03 | 0.56 |
| HbA1c (%) | 0.02 | 0.02 to 0.02 | <0.0001 | 0.001 | -0.01 to 0.01 | 0.75 |
| Systolic blood pressure (mmHg) | 0.27 | 0.17 to 0.37 | <0.0001 | -0.03 | -0.28 to 0.21 | 0.78 |
| Diastolic blood pressure (mmHg) | 0.42 | 0.37 to 0.48 | <0.0001 | 0.02 | -0.11 to 0.16 | 0.71 |
| HDL cholesterol (mmol/l) | -0.02 | -0.02 to -0.01 | <0.0001 | 0.01 | 0.001 to 0.01 | 0.01 |
| Triglycerides (mmol/l) | 0.04 | 0.03 to 0.04 | <0.0001 | -0.02 | -0.03 to 0.37 | 0.003 |
