# Supplementary material for: Defining Obesity Cut-Off Points for Migrant South Asians
Source: PLoS One. 2011 Oct 19;6(10):e26464. doi: 10.1371/journal.pone.0026464 (PMC3198431; doi:10.1371/journal.pone.0026464)
Supplement: Table S2 — Relationship between waist circumference (cm) and risk factors. (DOC) [file pone.0026464.s006.doc]

**Table S2**

**Relationship between waist circumference (cm) and risk factors**

|  | Total cohort | | | Ethnicity interaction (effect of South Asian ethnicity compared to White European) | | |
| --- | --- | --- | --- | --- | --- | --- |
|  | Coefficient | 95% Confidence Interval | P value | Coefficient | 95% Confidence Interval | P value |
| Fasting glucose (mmol/l) | 0.02 | 0.01 to 0.02 | <0.0001 | 0.002 | -0.003 to 0.01 | 0.48 |
| 2 hour glucose (mmol/l) | 0.03 | 0.03 to 0.04 | <0.0001 | 0.01 | 0.001 to 0.03 | 0.03 |
| HbA1c (%) | 0.01 | 0.01 to 0.01 | <0.0001 | 0.002 | -0.001 to 0.01 | 0.17 |
| Systolic blood pressure (mmHg) | 0.20 | 0.16 to 0.23 | <0.0001 | 0.10 | 0.003 to 0.20 | 0.04 |
| Diastolic blood pressure (mmHg) | 0.17 | 0.15 to 0.19 | <0.0001 | 0.05 | -0.01 to 0.10 | 0.08 |
| HDL cholesterol (mmol/l) | -0.01 | -0.01 to -0.01 | <0.0001 | 0.002 | 0.0004 to 0.004 | 0.02 |
| Triglycerides (mmol/l) | 0.02 | 0.02 to 0.02 | <0.0001 | -0.003 | -0.01 to 0.001 | 0.13 |
