# Supplementary material for: Defining Obesity Cut-Off Points for Migrant South Asians
Source: PLoS One. 2011 Oct 19;6(10):e26464. doi: 10.1371/journal.pone.0026464 (PMC3198431; doi:10.1371/journal.pone.0026464)
Supplement: Table S3 — White European equivalent cut-off point for South Asians for a BMI of 30 kg/m2 excluding those on antihypertensive or lipid lowering medication. (DOC) [file pone.0026464.s007.doc]

**Table S3. White European equivalent cut-off point for South Asians for a BMI of 30kg/m^2^ excluding those on antihypertensive or lipid lowering medication**

|  | Males | Females |
| --- | --- | --- |
| Lipid factor | 26.5 kg/m^2^ (22.1 kg/m^2^ to 30.8 kg/m^2^) | 23.4 kg/m^2^ (18.5 kg/m^2^ to 28.3 kg/m^2^) |
| BP factor | 27.7 kg/m^2^ (23.3 kg/m^2^ to 32.0 kg/m^2^) | 29.1 kg/m^2^ (23.0 kg/m^2^ to 35.2 kg/m^2^) |
