# Supplementary material for: Defining Obesity Cut-Off Points for Migrant South Asians
Source: PLoS One. 2011 Oct 19;6(10):e26464. doi: 10.1371/journal.pone.0026464 (PMC3198431; doi:10.1371/journal.pone.0026464)
Supplement: Table S4 — White European equivalent waist circumference (cm) cut-off points for South Asians excluding those on antihypertensive or lipid lowering medication. (DOC) [file pone.0026464.s008.doc]

**Table S4. White European equivalent waist circumference (cm) cut-off points for South Asians excluding those on antihypertensive or lipid lowering medication**

|  | Males  (White European = 102 cm) | Females  (White European = 88 cm) |
| --- | --- | --- |
| Lipid factor | 92.3 cm (80.4 cm to 104.1 cm) | 73.2 cm (63.1 cm to 83.4 cm) |
| BP factor | 97.0 cm (86.2 cm to 107.8 cm) | 86.6 cm (70.4 cm to 102.8 cm) |
