# Supplementary material for: Defining Obesity Cut-Off Points for Migrant South Asians
Source: PLoS One. 2011 Oct 19;6(10):e26464. doi: 10.1371/journal.pone.0026464 (PMC3198431; doi:10.1371/journal.pone.0026464)
Supplement: Table S5 — White European equivalent cut-off point for South Asians for a BMI of 30 kg/m2 excluding HbA1c from the glycaemia factor. (DOC) [file pone.0026464.s009.doc]

**Table S5. White European equivalent cut-off point for South Asians for a BMI of 30 kg/m^2^ excluding HbA1c from the glycaemia factor**

|  | Males | Females |
| --- | --- | --- |
| Glycaemia factor | 22·6 kg/m^2^ (20·7 kg/m^2^ to 24·5 kg/m^2^) | 21·5 kg/m^2^ (19·5 kg/m^2^ to 23·5 kg/m^2^) |
| Glycaemia factor without HbA1c | 23.4 kg/m^2^ (19.2 kg/m^2^ to 27.6 kg/m^2^) | 23.5 kg/m^2^ (18.5 kg/m^2^ to 28.6 kg/m^2^) |
