# Supplementary material for: Defining Obesity Cut-Off Points for Migrant South Asians
Source: PLoS One. 2011 Oct 19;6(10):e26464. doi: 10.1371/journal.pone.0026464 (PMC3198431; doi:10.1371/journal.pone.0026464)
Supplement: Table S6 — White European equivalent waist circumference (cm) cut-off points for South Asians excluding HbA1c from the glycaemia factor. (DOC) [file pone.0026464.s010.doc]

**Table S6. White European equivalent waist circumference (cm) cut-off points for South Asians excluding HbA1c from the glycaemia factor**

|  | Males  (White European = 102 cm) | Females  (White European = 88 cm) |
| --- | --- | --- |
| Glycaemia factor | 83·8 cm (79·3 cm to 88·2 cm) | 69·3 cm (65·2 cm to 73·4 cm) |
| Glycaemia factor without HbA1c | 86.1 cm (75.7 cm to 96.5 cm) | 73.9 cm (62.7 cm to 85.1 cm) |
